# Supplementary material for: Profiling of Human Neural Crest Chemoattractant Activity as a Replacement of Fetal Bovine Serum for In Vitro Chemotaxis Assays
Source: Int J Mol Sci. 2021 Sep 18;22(18):10079. doi: 10.3390/ijms221810079 (PMC8468192; doi:10.3390/ijms221810079)
Supplement: Supplementary file 1 [file ijms-22-10079-s001.zip › ijms-1331524-supplementary.pdf]

International Journal of
Molecular Sciences

MDPI

Article

Profiling of Human Neural Crest Chemoattractant Activity as a Replacement of Fetal Bovine Serum for In Vitro Chemotaxis Assays

Xenia Dolde [1], Christiaan Karreman [1], Marianne Wiechers [1], Stefan Schildknecht [2] and Marcel Leist [1,*]

[1] Department of Biology, University of Konstanz, 78457 Konstanz, Germany; xenia.dolde@uni-konstanz.de (X.D.); christiaan.karreman@uni-konstanz.de (C.K.); marianne.wiechers@uni-konstanz.de (M.W.)
[2] Department of Life Sciences, Albstadt-Sigmaringen University of Applied Sciences, 72488, Sigmaringen, Germany; Stefan.Schildknecht@uni-konstanz.de
* Correspondence: Email: marcel.leist@uni-konstanz.de; Tel.: +49 (0) 7531 88 5037; Fax: +49 (0) 7531 88 5039

A

Chemotaxis activity unit: 25% migration increase

$$\text{Specific activity } \left(\frac{U}{mg}\right) = \frac{\text{units/volume } \left(\frac{U}{\mu l}\right)}{\text{total protein/volume } \left(\frac{mg}{\mu l}\right)}$$

$$\text{Purification factor} = \frac{\text{specific activity of fraction } \left(\frac{U}{mg}\right)}{\text{specific activity of FBS } \left(\frac{U}{mg}\right)}$$

B

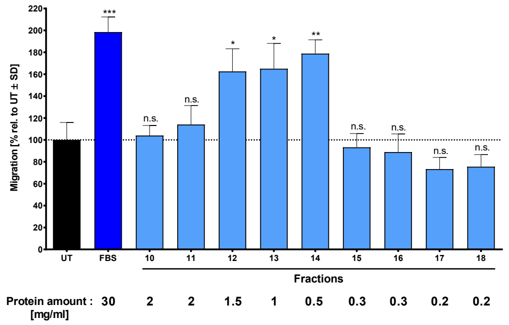

C

Description	Coverage	# Unique Peptides	MW [kDa]
Alpha-1-antiproteinase	50.0	28	46.1

D

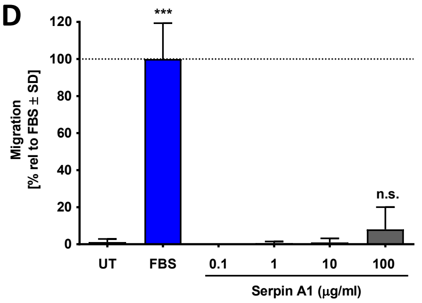

Figure S1. Characterization and purification of the chemotaxis-promoting factor of bovine serum and exclusion of serpin A1 as chemoattractant candidate. (**A**) Definition and calculation of chemotaxis activity unit, specific activity and purification factor. One unit is defined as the migration increase of NCCs in the cMINC assay within 24 h migration time, as well as in the NC-MT assay within 6 h migration time. (**B**) FBS was purified via acetone precipitation and two anion exchangers. Fractions of the second anion exchanger (HiTrap Q HP) were tested at a final concentration of 5% in the cMINC assay for their migration increasing activity. As positive control 5% FBS was used. UT: untreated. Data are normalized to untreated control and shown as means ± SD of two independent experiments. *: $p < 0.05$, **: $p < 0.01$, ***: $p < 0.001$, ns: not significant as determined by one-way ANOVA followed by Dunnett's post hoc test (compared to untreated control). (**C**) Fraction 14 of the second anion exchanger (HiTrap Q HP) indicated the highest migration increase. The fraction was separated on a 10% SDS gel and analysed by mass spectrometry (MS) analysis. The table represents the MS result of fraction 14 detecting serpin A1 as the most abundant protein. (**D**) The most abundant protein serpin A1 present in the fraction with the highest migration activity, was tested at the indicated concentrations in the NC-MT assay. Data are normalized to FBS and shown as means of five technical replicates ± SD. ***: $p < 0.001$, ns: not significant as determined by one-way ANOVA followed by Dunnett's post hoc test (compared to untreated control).

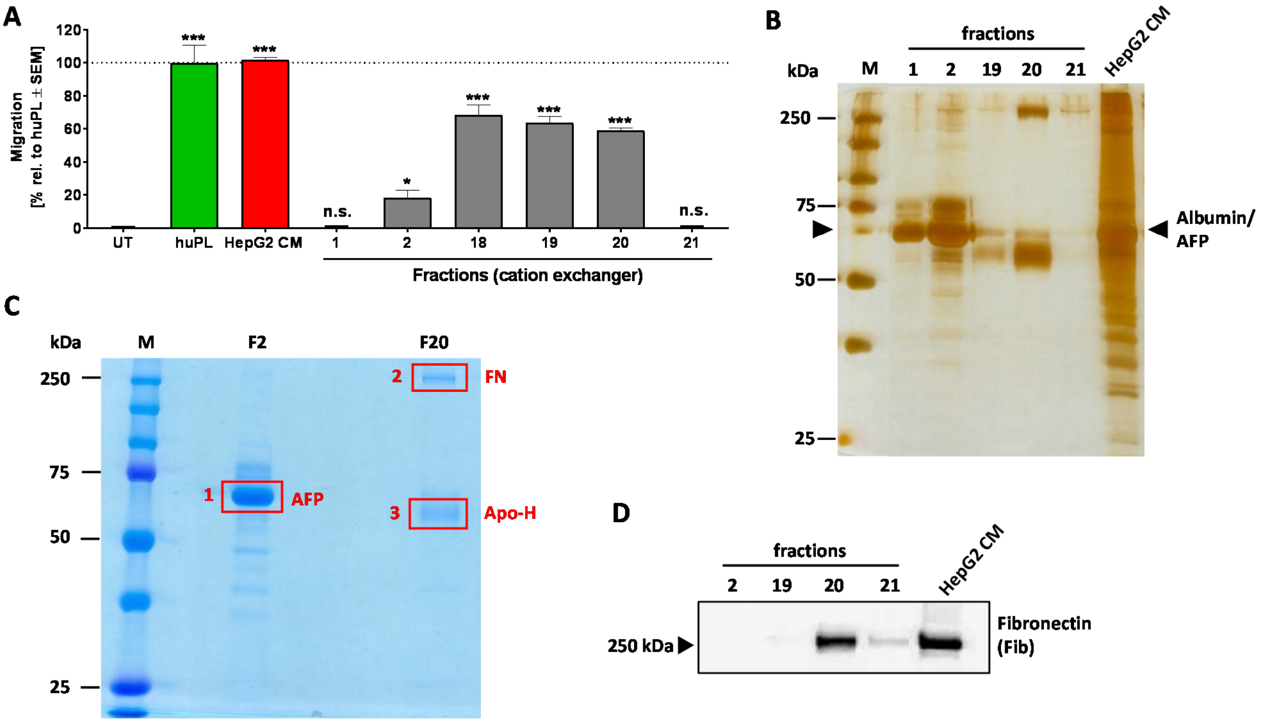

Figure 2. Characterization of chemotaxis-promoting factor in HepG2 CM after acetone precipitation and cation exchanger. (**A**) HepG2 CM was precipitated by acetone, followed by a cation exchange column (HiScreen Capto SP ImpRes). Fractions 1 and 2 (flow-through) and fractions 18–21 of the cation exchanger were tested at a final concentration of 20% in the NC-MT-HTS assay. As positive control 5% huPL and 100% HepG2 CM were used. UT: untreated. Data are normalized to 100% HepG2 CM and are shown as means ± SEM of two independent experiments *: p <0.05, ***: p < 0.001, ns: not significant as determined by one-way ANOVA followed by Dunnett's post hoc test (compared to untreated control). (**B**) Samples from HepG2 CM and fractions 1, 2, and 19–21 of the cation exchange column were separated on a 10% SDS gel, and bands were visualized by silver staining. The albumin/AFP band is indicated by arrow heads. (**C**) Samples of fraction 2 and fraction 20 of the cation exchanger were separated on a 10% SDS gel, and bands were visualized by coomassie staining. Bands cut out for MS analysis are marked in red. The most abundant proteins, detected in each band by MS analysis are stated. 1: Alpha-fetoprotein (AFP); 2: Fibronectin (FN); 3: Apolipoprotein-H (Apo-H). (**D**) Samples from HepG2 CM and fractions 2, 19, 20 and 21 of the cation exchange column (HiScreen Capto SP ImpRes) were separated on a 10% SDS gel and afterwards transferred onto a nitrocellulose membrane. Western blot analysis using an anti-fibronectin antibody was performed.

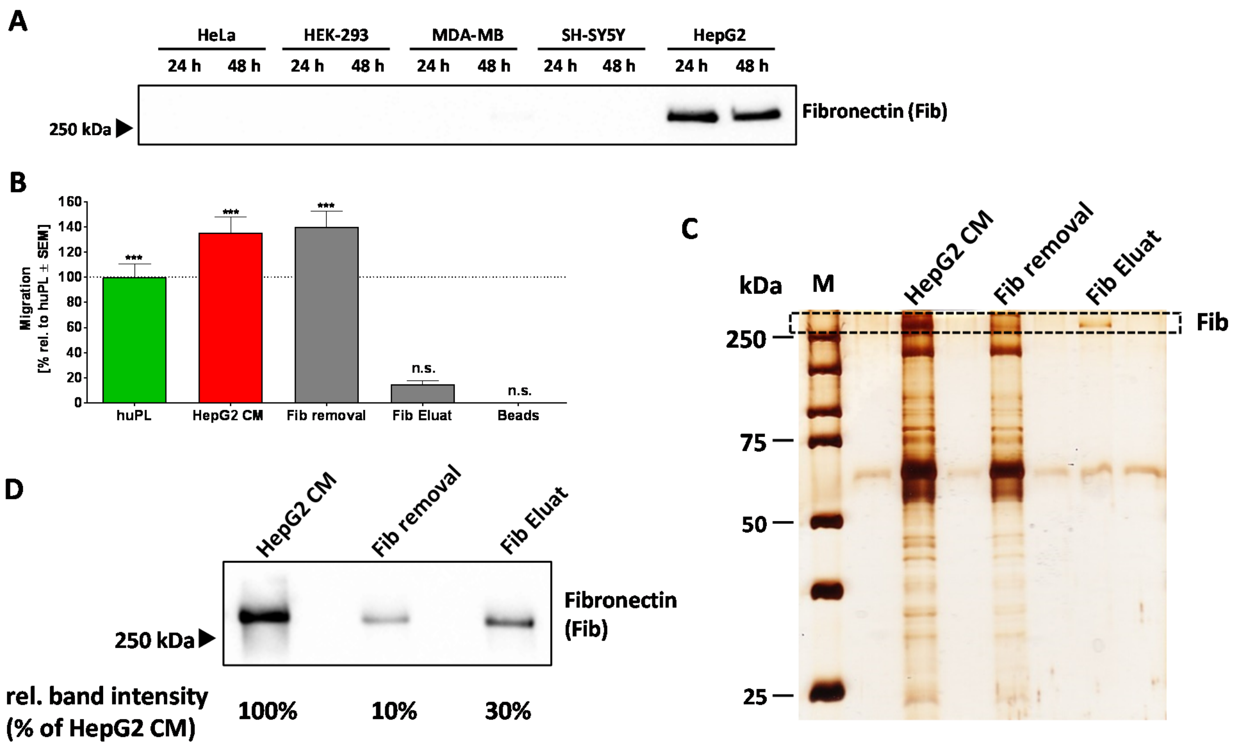

Figure 3. Exclusion of fibronectin as chemoattractant factor in HepG2 conditioned medium. (**A**) Conditioned medium was produced from the indicated cell lines. Samples were taken after 24 h and 48 h of starvation (including a mediumchange and washing step with PBS between 24 h and 48 h), separated on a 10% SDS gel, and transferred onto a nitrocellulose membrane. Western blot analysis using anti-fibronectin antibody was performed. (**B**) Gelatin Sepharose 4B beads (Cytiva) were mixed with 5 mg/mL BSA to block nonspecific protein binding, centrifuged at 100 x g for 3 min and the supernatant was discarded. This procedure was repeated with 6 M urea solution followed by two washing steps with PBS, all at room temperature. The washed gelatin beads were mixed with 10 mL of HepG2 CM and incubated for 24 h at 4 °C on a tube rotator. The next day the gelatin beads with the bound fibronectin were centrifuged at 100 x g for 3 min and the supernatant was transferred into a fresh tube. New gelatin beads were added to the HepG2 CM and incubated for another 24 h at 4 °C on a tube rotator. After centrifugation, the HepG2 CM without fibronectin (Fib removal) was transferred to a new tube and tested at a concentration of 100% in the NC-MT assay. The gelatin beads were washed with 6 M urea solution to remove the bound fibronectin and centrifuged at 100 x g for 3 min. The supernatant containing fibronectin (Fib Eluat) was centrifuged through a 10 kDa cut-off filter, and washed once with PBS to remove the urea solution, before it was tested at a final concentration of 20% in the NC-MT assay. Data are normalized to 5% huPL and shown as means ± SEM from three independent experiments. ***: $p < 0.001$, ns: not significant as determined by one-way ANOVA followed by Dunnett's post hoc test (compared to untreated control). (**C**) Samples from HepG2 CM before (HepG2 CM) and after fibronectin removal (Fib removal) as well as fibronectin eluated from the beads (Fib Eluat) were separated on a 10% SDS gel and bands were visualized by silver staining. (**D**) Samples from HepG2 CM before (HepG2 CM) and after fibronectin removal (Fib removal) as well as fibronectin eluated from the beads (Fib Eluat) were separated on a 10% SDS gel and afterwards transferred onto a nitrocellulose membrane. Western blot analysis using an anti-fibronectin antibody was performed.

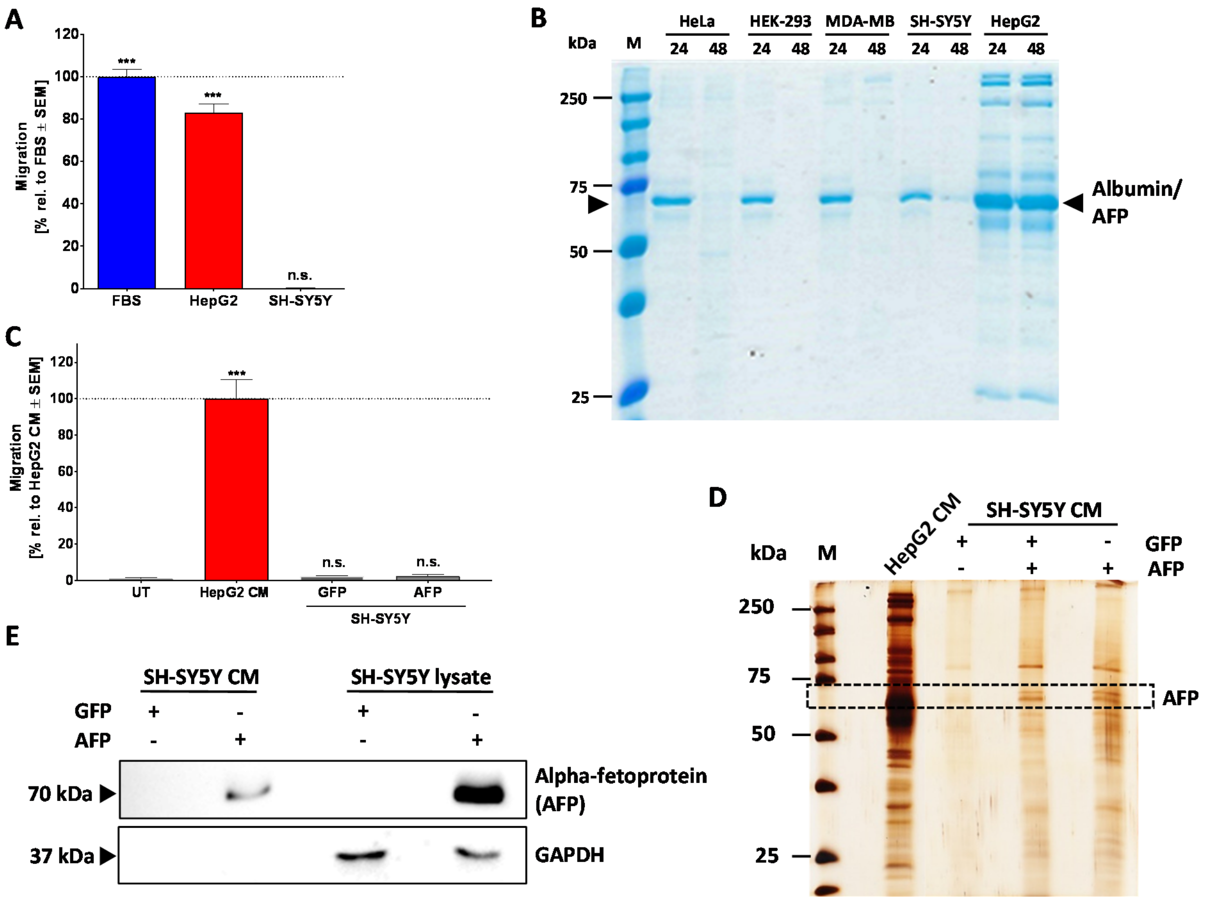

Figure 4. Exclusion of alpha-fetoprotein (AFP) as chemoattractant factor in HepG2 conditioned medium. (**A**) HepG2 and SH-SY5Y cells were starved with medium containing 0% FBS for 48 h. The conditioned medium was added at a concentration of 100% to the reservoirs of the NC-MT assay and NCCs were allowed to migrate for 6 h. Data are normalized to 5% FBS and shown as means ± SEM from three independent experiments. ***: $p < 0.001$, ns: not significant as determined by one-way ANOVA followed by Dunnett's post hoc test (compared to untreated control). (**B**) Conditioned medium was produced from the indicated cell lines. Samples were taken after 24 h and 48 h of starvation (including a mediumchange and washing step with PBS between 24 h and 48 h), separated on a 10% SDS gel, and bands were visualized by coomassie staining. (**C**) Cells were transfected with Lipofectamine™ 3000 (Thermo Fisher Scientific). Therefore, cells were seeded in 6-well plates and grown until they reached 70% confluency. For transfection, 5 µg DNA was mixed with Opti-MEM™ medium, Lipofectamine™ 3000 reagent, P3000™ reagent, and incubated for 15 min at room temperature, before the DNA-lipid complex was added to the cells. After 24 h of incubation, the medium was aspirated, fresh medium with 0% FBS was added, and the cells were incubated for another 24 h. The conditioned medium was harvested and centrifuged at 314 x g for 4 min, to remove cell debris. The alpha 1 fetoprotein clone IRATp970C1243D was obtained from Source BioScience Cambridge. Conditioned medium was tested in the NC-MT assay at a final concentration of 100%. UT: untreated. Data are normalized to 100% HepG2 CM and shown as means ± SEM from three independent experiments. ***: $p < 0.001$, ns: not significant as determined by one-way ANOVA followed by Dunnett's post hoc test (compared to untreated control). (**D**) Samples from SH-SY5Y CM of cells transfected with constructs containing AFP, GFP-control or both were separated on a 10% SDS gel, and bands were visualized by silver staining. (**E**) Samples from SH-SY5Y CM and lysate of cells transfected with constructs containing either AFP or GFP as control were separated on a 10% SDS gel, and afterwards transferred onto a nitrocellulose membrane. Western blot analysis using an anti-AFP and an anti-GAPDH antibody was performed.

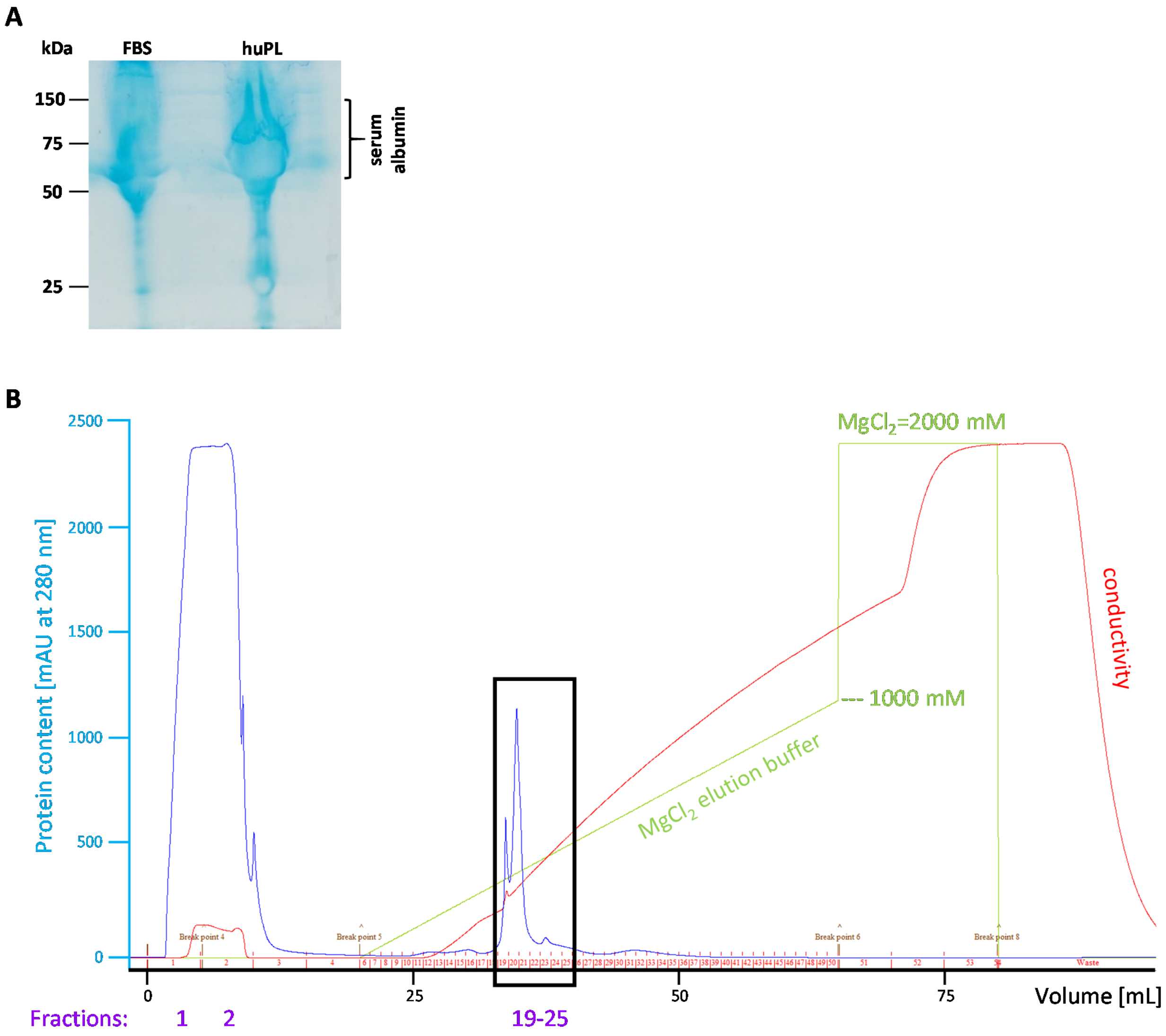

Figure 5. Characterization and purification of the chemotaxis-promoting factor in huPL. (**A**) FBS and commercial huPL (both 30 mg/mL) were fractionated by polyacrylamide gel electrophoresis (PAGE). Each lane was loaded with 20 µl of sample. For visualization of the protein, gels were stained with Coomassie. MW markers were run in parallel (lane not shown, but marker positions are indicated to the left). The large content of serum albumin, indicated by a smear at MW > 55 kDa, prevented separation and visualization of proteins with MW > 50 kDa. (**B**) Typical elution profile of huPL on a cation exchange column (HiScreen Capto SP ImpRes). The protein content is indicated in blue, the conductivity is shown in red and the concentration of the elution buffer ($MgCl_2$) is depicted in green. The numbers of the collected fractions are given at the bottom in violet. The column was loaded with 5 mL huPL and then washed with 20 mL (5 x the volume of the column) 10 mM Tris pH 7.4. All unspecifically bound proteins were washed off, shown in the first peak (fraction 1/2) together with a small salt peak. The elution started with a gradient of 1–1000 mM $MgCl_2$, followed by a high salt washing step with 2 M $MgCl_2$. Fractions 19–25 triggered chemotaxis in the NC-MT assay (black rectangle).

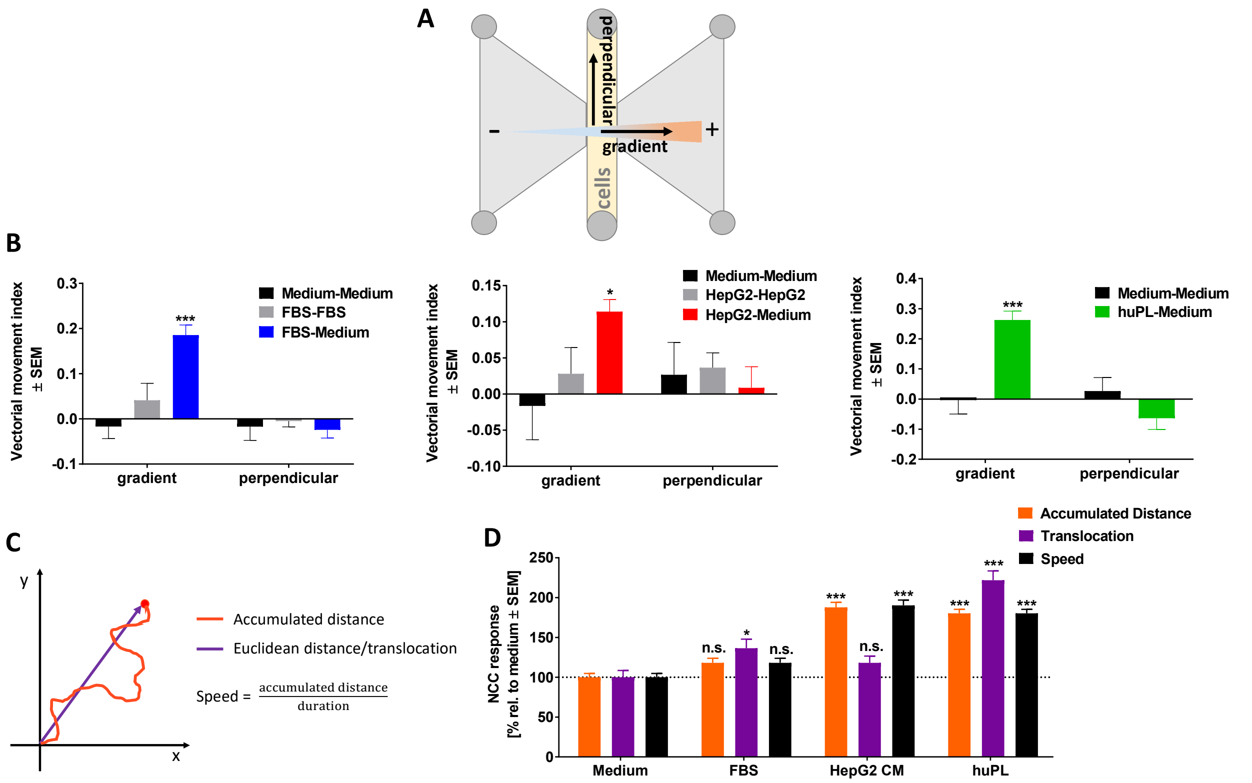

Figure 6. Single cell migration behaviour in µ-slide chemotaxis chambers. (**A**) Graphical representation of the vectorial movement index. This index can be parallel to the gradient (gradient) or perpendicular to the gradient (perpendicular). A chemotactic effect is fulfilled if gradient > perpendicular for the chemoattractant-medium (+/–) condition, whereas for the control conditions medium/medium (–/–) and chemoattractant/chemoattractant (+/+) both vectorial movement indices are around 0. (**B**) Calculation of vectorial movement index gradient and vectorial movement index perpendicular for FBS, huPL and HepG2 CM treated cells. Data are shown as means ± SEM of at least two independent experiments *: p <0.05, ***: $p < 0.001$, ns: not significant as determined by two-way ANOVA followed by Sidak's post hoc test (compared to Medium-Medium conditions). (**C**) Graphical representation and definition of accumulated distance, translocation (euclidean distance from start to endpoint of track) and speed (distance along the track per time). (**D**) Cells were treated with medium, FBS, huPL and HepG2 CM and time-lapse imaging was performed for 24 h. Accumulated distance, translocation and speed were calculated from the trajectory of 20 cells from two independent experiments. *: p <0.05, ***: $p < 0.001$, ns: not significant as determined by one-way ANOVA followed by Dunnett's post hoc test (compared to medium; performed separately for each endpoint).
